# Calibrating a Transformer-Based Model’s Confidence on Community-Engaged Research Studies: Decision Support Evaluation Study

**DOI:** 10.2196/41516

**Published:** 2023-03-20

**Authors:** Brian Ferrell, Sarah E Raskin, Emily B Zimmerman

**Affiliations:** 1 Virginia Commonwealth University Richmond, VA United States; 2 L. Douglas Wilder School of Government and Public Affairs Virginia Commonwealth University Richmond, VA United States; 3 Center on Society and Health Virginia Commonwealth University Richmond, VA United States

**Keywords:** explainable artificial intelligence, XAI, Bidirectional Encoder Representations From Transformers, BERT, transformer-based models, text classification, community engagement, community-engaged research, deep learning, decision support, trust, confidence

## Abstract

**Background:**

Deep learning offers great benefits in classification tasks such as medical imaging diagnostics or stock trading, especially when compared with human-level performances, and can be a viable option for classifying distinct levels within community-engaged research (CEnR). CEnR is a collaborative approach between academics and community partners with the aim of conducting research that is relevant to community needs while incorporating diverse forms of expertise. In the field of deep learning and artificial intelligence (AI), training multiple models to obtain the highest validation accuracy is common practice; however, it can overfit toward that specific data set and not generalize well to a real-world population, which creates issues of bias and potentially dangerous algorithmic decisions. Consequently, if we plan on automating human decision-making, there is a need for creating techniques and exhaustive evaluative processes for these powerful unexplainable models to ensure that we do not incorporate and blindly trust poor AI models to make real-world decisions.

**Objective:**

We aimed to conduct an evaluation study to see whether our most accurate transformer-based models derived from previous studies could emulate our own classification spectrum for tracking CEnR studies as well as whether the use of calibrated confidence scores was meaningful.

**Methods:**

We compared the results from 3 domain experts, who classified a sample of 45 studies derived from our university’s institutional review board database, with those from 3 previously trained transformer-based models, as well as investigated whether calibrated confidence scores can be a viable technique for using AI in a support role for complex decision-making systems.

**Results:**

Our findings reveal that certain models exhibit an overestimation of their performance through high confidence scores, despite not achieving the highest validation accuracy.

**Conclusions:**

Future studies should be conducted with larger sample sizes to generalize the results more effectively. Although our study addresses the concerns of bias and overfitting in deep learning models, there is a need to further explore methods that allow domain experts to *trust* our models more. The use of a calibrated confidence score can be a misleading metric when determining our AI model’s level of competency.

## Introduction

### Background

In community-engaged research (CEnR), academic researchers partner with communities to improve research and community outcomes. Effective CEnR requires prolonged community engagement and access to resources such as funding and training. An ability to track the types and number of CEnR studies taking place at universities can support collaboration by helping to identify partnerships, fostering communication, and sharing results. It can also support institutional needs such as planning, reporting, and accountability. Our goal is to identify CEnR studies by using institutional review board (IRB) protocols available through a university IRB database.

In addition to classifying whether studies qualify as CEnR, this project aimed to classify the level of collaboration with community partners in the research process. This is important because classifying different levels of CEnR can allow us to see whether researchers are deeply engaged with the community partner or whether the engagement is primarily focused on instrumental aims such as accessing data or recruiting participants [1,2]. Providing estimates of the level of engagement within CEnR could help a university plan for infrastructure needs and provide more accurate reporting. Because of CEnR’s call-for-action approach, classifying a study based on the partner’s level of engagement allows the action research to be more transparent. Organizations participating in research in a way that uses dissemination to the community, engagement in fieldwork, appropriate design, and development of solutions iteratively alongside a principal investigator make the solutions they are presenting more transparent (ie, more dependable, transferable, trustworthy, and workable [3]).

Virginia Commonwealth University began tracking CEnR studies in 2013 because of the aforementioned important principles. CEnR studies were labeled under 3 custom fields in the university IRB’s web-based human participants protocol submission form, as part of an award from the National Center for Advancing Translational Sciences [4]. Issues arose with these custom fields concerning the quality of how CEnR studies were labeled [5]. Principal investigators, when submitting a protocol to the IRB, were asked to list whether there were any community partners in the proposed research study and (if yes) to describe their role by choosing one of the following descriptions:

1. Community partners only provide access to study subjects or project sites. They are not involved with study design, subject recruitment, data collection, or data analysis.
2. Community partners do not make decisions about the study design or conduct but provide guidance to the researcher about the study design, subject recruitment, data collection, or data analysis.
3. Community partners make decisions with the researcher(s) about the study’s research activities and/or help conduct those activities (i.e., study design, subject recruitment, data collection, and/or data analysis).

Some issues that arose when entering data into these custom fields were identified in 2018, for example, inconsistent interpretation of the role of a community partner by principal investigators or study administrators not realizing that their work qualifies as CEnR. These concerns led to an exploratory study, where we hand labeled a set of 280 study protocols from the IRB database on 3-level and 6-level classification spectra [6]. We built a prototype model by comparing traditional deep learning (DL) methods with those of transformer-based models. The aim of the study was to create a proof-of-concept model by using transformer-based models to test an automated methodology of tracking CEnR studies using protocols submitted to the university’s web-based IRB system. Using numerous comparisons in this pilot study, we found that transfer learning had superior performance compared with traditional DL models. The work presented in the paper led our team to take a closer look at these previously trained models and improve upon them by using fine-tuning methods [7] (eg, different learning rates for different layers and layer freezing). In the initial experiments, we found that models generalized better when categorizing 3 classes rather than 6; therefore, the models used in the new experiments were trained on 3 classes. By conducting experiments to improve our prototype models, we found that transformer models performed better when their learning rates differed among 4 layer groups. These results substantially improved upon prior experimentations, leading to the next phase of performance testing. The research question we wished to explore involved evaluating these models in different ways to see whether we can trust their predictions.

Our paper proceeds as follows. First, we review the literature comparing human-level performances with those of DL models, as well as novel evaluation research methods and tools. Although our metrics are not necessarily performance related because the *correct* answers are subject to our opinion, these papers were important to our research because they helped to create a framework for rigorously evaluating models as well as attempting to explain them. Next, we describe our data classes, methods, and models trained. Finally, we report our findings by presenting *F*_1_-scores, accuracies, confusion matrices, and confidence scores of our models and conclude by considering the challenges and implications for future work.

### Related Work

#### Designing Evaluation Approaches

The idea behind designing evaluation approaches is to explain or explore model predictions, which allows us humans to improve decision-making, as well as create evidence for designing solid decision-making systems. Mucha et al [8] evaluated an artificial intelligence (AI) model’s effect on human decision-making by giving participants the task of estimating the final grade of a student based on a list of attributes. The participants were then presented with advice from the decision support system, and, based on this advice and added information, they were given the opportunity to change their estimation. The study answered an important question: “Did people adhere to the computer’s advice or not?” The authors state, “At the center is the question how the way we represent machine behavior and reasoning as interfaces, i.e. specific design elements, affects human decision making and behavior.” Although our study does not have a decision-making support system, we are comparing our predictions with those of our model to assess concordance and learn about techniques that can be a viable option in terms of influencing us.

Bansal et al [9] reported using AI as a complementary decision maker rather than the sole one. The paper motivated us to view our model as a *fourth reviewer*, rather than solely relying on its predictions, which may improve decision-making performance. Bansal et al [9] conducted 2 types of experiments using sentiment analysis and question answering. They used different combinations of explanation strategies, such as showing a model’s predictions and confidence, highlighting influential words, color-coding positive and negative words, or showing the top 2 predicted classes. The authors presented results of the performances of each experiment as well as how each explanation strategy influenced a person’s decision-making process. In explaining the differences among the accuracies of the AI system versus human versus AI-human teams, they mention that, with AI explanations, people tend to repose too much trust in the AI system even when it is “worryingly” incorrect. The study highlights the consequences of people blindly trusting AI even when it is wrong, rather than using their own discretion and using an appropriate amount of reliance on the AI system. This points to the importance of using domain experts when comparing predictions because experts in a particular field may be less likely to defer to AI as easily as nonexperts (eg, participants from Amazon Mechanical Turk). The authors mention the need for new interaction methods to increase this synergetic complementary performance beyond simply showing an AI system’s confidence; nonetheless, we present confidence scores in our study to find a pattern or threshold between correct predictions with high confidence and incorrect predictions with low confidence.

This coincides with the aims of the study by Chromik et al [10], who considered how people without technical expertise attempt to understand a model’s behavior by creating mental models that reflect their belief about how a system works. The authors examined whether these nontechnical users are prone to an illusion of explanatory depth (IOED) [11]. IOED refers to people forming an inaccurate understanding of complex systems combined with overconfidence about how they perceive it. Rozenblit and Keil [11] argue that most people feel that they understand the world in far greater detail, coherence, and depth than they really do. The authors break down IOED into 4 features, 2 of which are potentially relevant to AI models (“representation/recovery confusion” and “label-mechanism confusion” through subtle interactions). As Chromik et al [10] state, users with certain insights into AI models might get the impression that they understand why a model makes a prediction for all observations. This is false because these black box models have features that interact in many ways (ie, some features may have a heavy influence on some observations but not on others). The study demonstrates how we must be mindful of these AI systems when deploying them in our organizations, societies, universities, corporations, and so on, to support nontechnical users by appropriately modeling the behavior of machine learning (ML) and DL model decisions.

The studies reviewed illustrate how AI models powerfully influence people’s decision-making abilities, which may result in blindly following a model’s predictions and overestimating one’s understanding of a model’s behavior. Open dialogue about the production of these models needs to be prevalent in all settings. As Dhanorkar et al [12] state, AI models must be closely examined, tested, and reviewed, and they need to be aligned with domain knowledge and social reality. The way to achieve this is by ensuring that domain experts remain in the loop to lead models toward correct explanations [13]. It is a challenge to explain model predictions, but a slow, evaluative process in each of the stages within the AI lifecycle, carried out in collaboration with domain experts, is a professional, transparent, and ethical step in the right direction.

#### Examples of Comparison Papers

It is said that AI will transform how life on this planet is currently shaped, whether that be with regard to stock trading [14], diagnosing patients [15], manufacturing [16], drug discovery [17], or even poetry [18]. In fact, Grace et al [19] report on survey findings from 400 ML researchers that indicated strong belief about the progression of AI and showed an aggregate forecast of the individual responses suggesting that there is a 50% chance that unaided machines can accomplish every task better and more cheaply than human workers within 45 years. Whether or not this forecast is accurate, AI is indeed progressing quickly. Most of the respondents said that the field of ML has accelerated more in the second half of their careers than the first half. With that being said, in the following paragraphs, we provide some examples of studies where AI might be close to exceeding human performance already, and others where humans are still the best. All these studies have limitations regarding, as well as insights into, how to perform well-designed and well-executed predictive modeling.

Blohm et al [20] provide a much-appreciated exhaustive evaluation study on text classification tasks, comparing automated ML (AutoML) with human data scientists. The authors examined 4 popular AutoML tools on 13 text classification data sets and found that, in 9 out of these 13 experiments, the best AutoML tool could not beat human-level performance. Although we will not go over these 13 data sets here, it was concluded that AutoML is a relatively new field in which there is a lot of potential to make AutoML tools increasingly useful and sophisticated, specifically with regard to investigating the changes produced by, and outcomes of using, various preprocessing techniques. Enos et al [21] compared the performances of humans and machines in a task involving deceptive speech detection and found the ML results to be promising. The authors interestingly point out that, when asked to judge whether something was true or a lie, individual differences (personality factors) had a huge impact on their success. This leads us to believe that the type of participant matters considerably when comparing their performance with that of an AI model. As is the case with surveying people and reporting on the results, certain biases must be considered when comparing humans with AI models. Other studies that have compared DL and ML models with human graders for diabetic retinopathy screening [22] and pigmented skin lesion classification [23] point out how concerning false negative cases can be. It is important to be mindful of what sort of impacts AI models have, for example, missing potential treatment referrals that would create risks of vision loss. However, Limwattanayingyong et al [22] point out that the DL models had a better false negative rate than the human graders. The thought of AI models making clinical decisions sounds frightening, but, when handled properly, AI models can reduce overhead costs (decreasing false positives) and improve treatment recommendations.

AI models do have certain weaknesses, such as when trained in low-resource settings [24] or under visual distortions [25,26]. Although AI model performances can be on par with those of humans, certain challenges may drastically alter performance. One thing to note is that models need to be able to perform at a high level when they are presented with real-world situations, meaning that sometimes the data presented look ugly (eg, not perfectly cleaned up). Therefore, exploring what these models are terrible at is a great way to improve the robustness of their learning systems. A strength of AI models is that they never get tired. The fatigue that comes with labeling data and making predictions puts human performance at risk, which may be something else to be cognizant of if one is labeling one’s own training data.

AI offers considerable promise in the aforementioned fields. Liu et al [27] point out that when carrying out these comparison studies, it is necessary to highlight these comparisons using out-of-sample (validation) data sets, which is what we have done. The authors state that comparison studies need to minimize bias as well as be thoroughly and transparently reported. In their systematic review of 122 full-text articles comparing DL algorithms with health care professionals for medical imaging tasks, they state, “These image repositories are rarely quality controlled for the images or their accompanying labels, rendering the DL model vulnerable to mistakes and unidentified biases. Population characteristics for these large data sets are often not available (either due to not being collected, or due to issues of accessibility), limiting the inferences that can be made regarding generalizability to other populations and introducing the possibility of bias toward particular demographics.” There is much uncertainty regarding the diagnosing performances of AI models, and there must be an emphasis on ethical and transparent reporting.

## Methods

### Overview

We examined 3 previously trained and tested transformer-based models and compared their predictions with those of 3 domain experts on a new validation data set of 45 research studies to test the generalizability on unseen data as well as to test whether they emulate our classification spectrum. In this section, we go over where the data came from, how the data were sampled, our reviewing process, and the saved models from previous experiments.

### Data

#### Categories Used to Label Protocols

Textbox 1 shows the 6 categories we originally used to label protocols [28]. These categories are based on the differences in the levels of community partner involvement in research that we observed in the IRB data set protocols. Although 6 classifications captured the observed levels of CEnR, this level of detail was not easily generalizable to train models on; therefore, we combined some of the classes together to fit a 3-level classification spectrum instead: we combined classes 1 and 2 (=1), as well as classes 3, 4, and 5 (=2), and kept class 0 as is.

**Community-engaged research (CEnR) levels that were used to manually classify the training data.**

- 0=no CEnR: research without a partnership or community engagement
- 1=non-CEnR partnership: there is reference to a partnership, but the relationship is either uncategorizable (eg, not adequately described) or not a traditional community-engaged partnership (eg, contractual relationship)
- 2=instrumental partnership: community partner primarily facilitates access to the *inputs* needed to conduct the study (eg, posting recruitment flyers, providing participant contact information, extracting data, and providing study sites for observation)
- 3=academic-led partnership: minimal yet important interaction between the research team and the community partner, which is often essential to project success (eg, academic partners take the lead on study design and research activities, with community partner involvement at particular points, such as troubleshooting recruitment or facilitating community meetings)
- 4=cooperative partnership: shared investment and mutual consideration between the research team and the community partner without shared decision-making (eg, community advisory boards that provided input on study design and methodology, reviewed data collection instruments, interpreted findings, and informed dissemination plans)
- 5=reciprocal partnership: community partners and research teams share decision-making power and governance (eg, community-based participatory research, team science, and steering committees with decision-making power)

#### Sampling of the Validation Set

The 45 studies were pulled from an unlabeled 6K IRB data set. We were not able to perform a stratified sampling method for each class because there was no way of knowing what class a study fit into. In previous experiments, we have made predictions on this 6K data set from our trained models on both our 3-level and 6-level classification spectra, but there is no way of knowing whether these predictions were correct. In addition, by combining our classes into a 3-level classification spectrum, although this is more accurate, it makes it so that we cannot know for sure whether we are collecting studies in which there is an academic-led partnership, consulting partnership, or reciprocal partnership because these are all combined into class 2. A way to work around this was looking at how we labeled CEnR levels (Textbox 1) and finding the top words for each class that we had already labeled by using the *Gensim* package in Python. We understand that this may cause sampling bias or an inaccurate representation of the population data set; hence, this can be considered a limitation of this study. We carried this out by searching for studies with relevant keywords that would typically show up in specific classes; for example, words such as “community-based participatory research,” “community partner,” “advisory board,” and “academic-community partnerships” would typically be found in classes 3 to 5, and “flyer,” “contract,” “recruitment,” and “client” would be found in class 1 or class 2. When the keyword search for a study was reported as “true” (meaning that it had a certain amount of these top associated words), we randomly pulled it. Regardless of the sampling method used, we are still labeling the studies ourselves and checking how the models label them.

### Reviewers and Models

#### Overview

This section reviews how we labeled our data set and provides a brief description of the models we compared with us. These models underwent what is called “transfer learning,” which is the use of unsupervised algorithms that are pretrained on larger data sets of unlabeled samples and then reused for another task such as ours. One imports these pretrained models and then retrains and evaluates them based on one’s own classifications.

#### Reviewers

There were 3 reviewers, 2 of whom have extensive experience in CEnR practices, whereas the third reviewer’s main role has revolved around leading in the development of the models. Each reviewer read and labeled the 45 studies as one of the 3 classes without seeing the predictions made by the algorithms or the other reviewers. The reviewers provided a rationale and reasoning for some studies to explain why they believed that the study fell into a particular class, whereas for other studies, the reviewers had questions or expressed confusion regarding what they thought the class should be. To address disagreements or confusion over protocols, once the reviewers had completed making their predictions, we first used comments in Google Sheets to respond to the disagreements and then met over Zoom (Zoom Video Communications, Inc) to reconcile any remaining differences in our predictions. There was a need to have our predictions agreed upon because we are comparing the models with us as a team rather than as individuals. We were left with 13 class 0 studies (29%), 13 class 1 studies (29%), and 19 class 2 studies (42%) in this validation data set (N=45).

#### Models

##### Bidirectional Encoder Representations From Transformers

Bidirectional Encoder Representations From Transformers (BERT) was introduced by Devlin et al [29]. BERT was pretrained on BookCorpus (800 million words) and Wikipedia (2500 million words), and owing to its 12 attention heads and 110 million parameters, it ensures state-of-the-art results on a variety of tasks. BERT has 2 main versions; we used the baseline version in our study. This pretrained model reads entire sequences of words (tokens) to learn a word’s contextual meaning. This is done through masking (hiding) words and attempting to predict the original word by looking at the context and the unmasked words around the hidden word, as well as through next-sentence prediction: predicting which sentence comes after a particular sentence, corresponding to the original text. These strategies were trained together during the pretraining phase; subsequently, to fine-tune BERT for our task, it was trained in a similar fashion but with a classification layer on top.

##### Bio+Clinical BERT

BERT is pretrained on data sets such as BookCorpus and Wikipedia, and this can be considered a general language model; however, Alsentzer et al [30] studied ways to improve upon this by using BERT models specifically pretrained with clinical text and discharge summaries. The authors used data from the Medical Information Mart for Intensive Care-III database to create 2 BERT models for clinical text: Clinical BERT, which contains all note types, and Discharge Summary BERT, which only contains discharge summaries, so that fine-tuning tasks such as ours can be better accomplished because our data sets contain clinical texts. They then trained the 2 BERT models on the clinical text: one initialized from the BERT base model and the other initialized from BioBERT [31] (this is the model we chose).

##### Cross-lingual Language Model-Robustly Optimized BERT Pretraining Approach

Cross-lingual Language Model-Robustly Optimized BERT Pretraining Approach (XLM-RoBERTa) was introduced by Conneau et al [32] in 2019 and updated in 2020. This model closely resembles the Robustly Optimized BERT Pretraining Approach architecture [33], except that it is a cross-lingual model, pretrained on 100 different languages. This type of model was made for cross-lingual transfer learning tasks and is trained on >2 terabytes of the Common Crawl corpus. It differs from BERT in terms of its tokenization and masking pattern, thus making it an interesting model with which to compare BERT.

### Calibration Scores

The final output from making predictions is often seen as a measure for how *confident* a model is in its predictions owing to the softmax values being predicted probabilities that add up to 1 (implying that the largest probability is for whatever class the model will predict). However, these outputs should not be used as true probabilities, mainly because the values are often too high and need to be calibrated [34-36]. To calibrate these values to reflect true confidence, we tried histogram binning [37] and temperature scaling [35], as well as other methods provided by the *NetCal* package in Python [38]; in addition, we used ensemble of near isotonic regression [39] for all 3 models because this had the lowest calibration error (with N=3 bins) using the average calibration error metric [40]. These methods were used mostly for experimental purposes; it does not imply that they are the best ways to calibrate our models, and other authors who wish to perform calibrations should conduct research into how to implement the aforementioned methods.

### Details From Our Previous Study

In our previous study [7], the models were trained using the Simple Transformers library, created by Rajapakse [41], which trains and evaluates transformer models (derived from the Hugging Face website) with fewer lines of code built on top of PyTorch. These models were trained on a single graphics processing unit device (NVIDIA GeForce RTX 2070 with 8 GB graphics double data rate 6 memory). For inference, we used an Intel Core i7-10750H central processing unit (2.60 GHz) with 32 GB RAM. Every model had weights corresponding to one of the 3 classes so that it was equally balanced during the training, ensuring that no class was heavily favored. When we trained these models, the training data set of 2028 samples comprised 614 (30.28%) class 0 samples, 645 (31.8%) class 1 samples, and 769 (37.92%) class 2 samples. The testing data set of 279 samples comprised 82 (29.4%) class 0 samples, 51 (18.3%) class 1 samples, and 146 (52.3%) class 2 samples. The holdout data set of 80 samples comprised 17 (21.3%) class 0 samples, 27 (33.8%) class 1 samples, and 36 (45%) class 2 samples. For evaluating these models, we compared both accuracy and *F*_1_-scores. After every model was trained, we made predictions on the holdout data set and recorded the accuracy, *F*_1_-score, and class accuracies. These previous experiments ensured that we were using the best performing models for this study, which will be used for comparison in this study with the new data set.

### Ethical Considerations

This study involved a secondary analysis of human participant protocols and therefore did not need an IRB review. The research and ethics protocols presented in this study were approved by the IRB of Virginia Commonwealth University, who provided these protocols for us to analyze. These studies are anonymous and private with regard to the public; we only worked with the IRB applications and not the research itself.

## Results

### Superiority of BERT

Table 1 shows results from all 3 text classification transformer-based models on the 45 samples of data derived from the IRB database. We present *F*_1_-scores and accuracies to show how they differ. Clearly, BERT outperforms Bio+Clinical BERT and XLM-RoBERTa by 11% on accuracy and by approximately 10% to 12% on the *F*_1_-score. It was confusing to see that it was not Bio+Clinical BERT that was outperforming the other models, considering that in previous experiments it had the highest accuracy and *F*_1_-score. This shows that different data sets produce different results; however, one certainty is that BERT provides a generalizable model based on these results as well as those of past experiments, proving its value.

**Table 1:** Results from the 3 models.

Model	Accuracy	*F*_1_-score
BERT^a^	0.6444	0.5928
Bio+Clinical BERT^b^	0.5333	0.4706
XLM-RoBERTa^c^	0.5333	0.4941

^a^BERT: Bidirectional Encoder Representations From Transformers.

^b^Bio+Clinical BERT: Bidirectional Encoder Representations From Transformers for Biomedical Text Mining+Clinical Bidirectional Encoder Representations From Transformers.

^c^XLM-RoBERTa: Cross-lingual Language Model-Robustly Optimized Bidirectional Encoder Representations From Transformers Pretraining Approach.

### Confusion Matrices, Precision, and Recall

**Figure 1:**
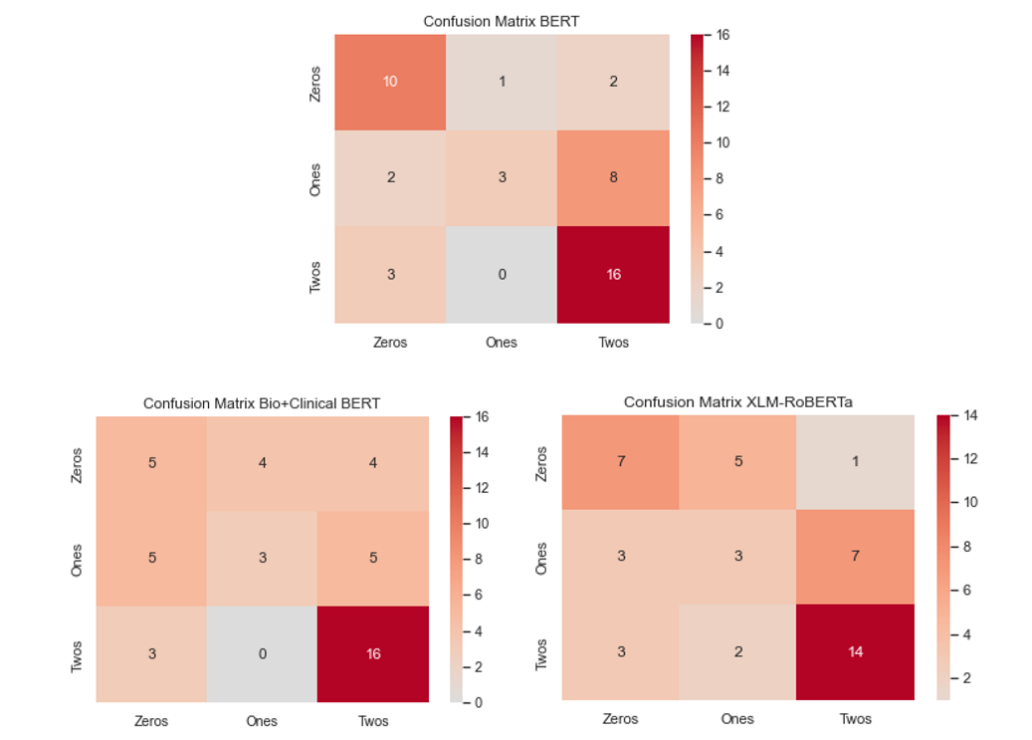
Confusion matrices for models. BERT: Bidirectional Encoder Representations From Transformers; Bio+Clinical BERT: Bidirectional Encoder Representations From Transformers for Biomedical Text Mining+Clinical Bidirectional Encoder Representations From Transformers; XLM-RoBERTa: Cross-lingual Language Model-Robustly Optimized Bidirectional Encoder Representations From Transformers Pretraining Approach.

Figure 1 shows the confusion matrices for all 3 predictions made by the transformer models. Confusion matrices are widely used and are helpful for visualizing errors for multiclass classification problems. The numbers that go diagonally from left to right indicate what the models correctly predicted, and the off-diagonal numbers indicate what the models labeled a class (y-axis) incorrectly as (x-axis); for example, the confusion matrix for BERT labeled 8 samples of class 1 incorrectly as class 2 (middle-right square). As we will see later, each model confused class 1 samples with class 2 samples the most. These classes are semantically close to each other (both have community partners) and offer very minor nuances in their sequences. Each model only got 3 samples of class 1 correct, and most of the correct predictions were from class 2. BERT and Bio+Clinical BERT also never misclassified a class 2 sample as a class 1 sample, but XLM-RoBERTa did so on 2 occasions. We further report on these confusion matrices in the following paragraph showing precision and recall for each class.

In Table 2, we show precision, recall, *F*_1_-score, and support (count of each class) for each class, as well as the averages of these scores for each model. The only values that do not have the average shown are the *F*_1_-scores because these have been presented in Table 1. It is important to look at precision and recall when one’s data sets are highly or even slightly imbalanced because accuracy is not always going to be a great measure for assessing performance (the *F*_1_-score provides a solid representation of how well a model performs on both precision and recall, making it meaningful as well). Recall is the number of true positives divided by the number of true positives plus the number of false negatives (number of correct predictions divided by support). A higher score for recall indicates a low false negative rate; therefore, for class 0, recall is high for BERT and decent for XLM-RoBERTa but low for Bio+Clinical BERT, indicating that Bio+Clinical BERT has a higher false negative rate for class 0. Each model had the same recall for class 1 (0.2308), which is the highest false negative rate; however, for class 2, each model had the highest recall values (high true positive rate) compared with those for the other classes. By contrast, precision is the number of true positives divided by the number of true positives plus the number of false positives. In other words, it is the number of correctly predicted values divided by the number of times a model incorrectly predicted something as a particular class; for example, in Figure 1, BERT correctly predicted 10 of the samples as class 0 but incorrectly predicted a class as 0 on 5 occasions; thus, 10 divided by 15 (10+5) is 0.6667, which can be seen in Table 2 as the precision for class 0. Whether precision is more important than recall or vice versa depends on the data and the problem. Our interpretation of these results indicates that our models struggled considerably with correctly predicting class 1 (low recall) and that the BERT model was more precise (returning more relevant results than irrelevant ones) and returned the highest number of relevant results.

**Table 2:** Classification report of confusion matrices.

Model and class		Precision	Recall	*F*_1_-score	Support
**BERT^a^**					
	0	0.6667	0.7692	0.7143	13
	1	0.7500	0.2308	0.3529	13
	2	0.6154	0.8421	0.7111	19
	Average	0.6774	0.6140	—^b^	—
**Bio+Clinical BERT^c^**					
	0	0.3846	0.3846	0.3846	13
	1	0.4286	0.2308	0.3000	13
	2	0.6400	0.8421	0.7273	19
	Average	0.4844	0.4858	—	—
**XLM-RoBERTa^d^**					
	0	0.5385	0.5385	0.5385	13
	1	0.3000	0.2308	0.2609	13
	2	0.6364	0.7368	0.6829	19
	Average	0.4916	0.5020	—	—

^a^BERT: Bidirectional Encoder Representations From Transformers.

^b^Not available.

^c^Bio+Clinical BERT: Bidirectional Encoder Representations From Transformers for Biomedical Text Mining+Clinical Bidirectional Encoder Representations From Transformers.

^d^XLM-RoBERTa: Cross-lingual Language Model-Robustly Optimized Bidirectional Encoder Representations From Transformers Pretraining Approach.

### Calibrated Scores

In this section, we examine the use of the calibrated confidence scores and explore whether there is some sort of threshold on the models correctly predicting a class as well as their level of average confidence. Tables 3-5 show the average confidence score for all 3 classes and each individual class for when the model correctly predicted a study and when it incorrectly predicted a study.

**Table 3:** Average confidence scores from calibrations for Bidirectional Encoder Representations From Transformers.

Class and predictions		Confidence score (%), average (SD)	Predictions (%)
**0, 1, and 2**			
	Correct	65.17 (11.52)	64.44
	Incorrect	62.19 (17.15)	35.56
**0**			
	Correct	60.45 (9.49)	76.92
	Incorrect	56.69 (3.96)	23.08
**1**			
	Correct	77.68 (16.41)	23.08
	Incorrect	56.26 (10.94)	76.92
**2**			
	Correct	65.78 (10.56)	84.21
	Incorrect	87.46 (21.71)	15.79

**Table 4:** Average confidence scores from calibrations for Bidirectional Encoder Representations From Transformers for Biomedical Text Mining+Clinical Bidirectional Encoder Representations From Transformers.

Class and predictions		Confidence score (%), average (SD)	Predictions (%)
**0, 1, and 2**			
	Correct	67.3 (17.33)	53.33
	Incorrect	53.48 (14.07)	46.67
**0**			
	Correct	64.31 (7.57)	38.46
	Incorrect	43.35 (5.59)	61.54
**1**			
	Correct	73.08 (33.31)	23.08
	Incorrect	62.16 (15.44)	76.92
**2**			
	Correct	67.16 (16.95)	84.21
	Incorrect	51.55 (2.4)	15.79

**Table 5:** Average confidence scores from calibrations for Cross-lingual Language Model-Robustly Optimized Bidirectional Encoder Representations From Transformers Pretraining Approach.

Class and predictions			Confidence score (%), average (SD)		Predictions (%)
**0, 1, and 2**					
	Correct	69.93 (17.52)		53.33	
	Incorrect	52.87 (11.64)		46.67	
**0**					
	Correct	68.04 (14.57)		53.85	
	Incorrect	46.72 (7.77)		46.15	
**1**					
	Correct	74.8 (24.78)		23.08	
	Incorrect	53.29 (10.06)		76.92	
**2**					
	Correct	69.84 (18.53)		73.68	
	Incorrect	59.43 (16.22)		26.32	

It is clear that for BERT, there was no clear distinction between the confidence scores on incorrect and correct predictions for the average confidence scores of all 3 classes as well as class 0. In fact, 64% of the predictions were correct for BERT, and regarding this 64%, the average confidence was 65%; however, it was 62% confident even for incorrect predictions, which is why there is no distinct threshold. In addition, the average confidence level was higher for incorrect predictions for class 2 than for correct predictions. However, one does notice a higher confidence level for class 1 at 77.68% for correct predictions and 56.26% for incorrect predictions; therefore, one could conclude that the studies BERT correctly predicted for class 1 had a high confidence level, although it only got 23% of class 1 correct. We conclude that, based on the calibration techniques used for the BERT model, this did not help with evaluating its predictions and thus would ultimately trick human reviewers if this were used as a reference for labeling studies. Perhaps this means BERT got lucky in achieving the highest accuracy among the 3 models, or it could mean that to be more generalizable, the level of confidence has to decrease; for example, instead of being 100% confident in one’s predictions with lesser accuracy, one has 65% confidence but with higher accuracy.

With Bio+Clinical BERT, there was a distinction between high confidence with its correct predictions and low confidence with its incorrect predictions. The average confidence level for all classes was 67% for correct predictions and 53% for incorrect predictions. How one interprets these results is that one looks at the prediction column to see the model’s accuracy for all 3 classes or each individual class, and of these correct predictions, one looks at the average confidence score that the model obtained; for example, Bio+Clinical BERT correctly predicted 84% of class 2, and regarding this 84%, it had an average confidence score of 67% as opposed to an average confidence score of 43% for the incorrect predictions. This tells us that of the predictions Bio+Clinical BERT got correct, it had a much higher confidence level than for the ones it got incorrect, ensuring that these calibrated outputs would allow a reviewer to have more *trust* in a model’s confidence (ie, using its confidence level as a valuable tool for labeling studies).

XLM-RoBERTa shows us another example of a large difference between high and low confidence scores. The average confidence score for correct predictions on all 3 classes was higher than that of the other 2 models; in fact, this model had the highest confidence scores for correct predictions for all the individual classes except for class 1 (74.8% vs BERT’s 77.68%). These types of results assure us that when XLM-RoBERTa’s confidence is 69% on average, we can trust that it has a higher probability of being correct.

## Discussion

### Principal Findings

**Figure 2:**
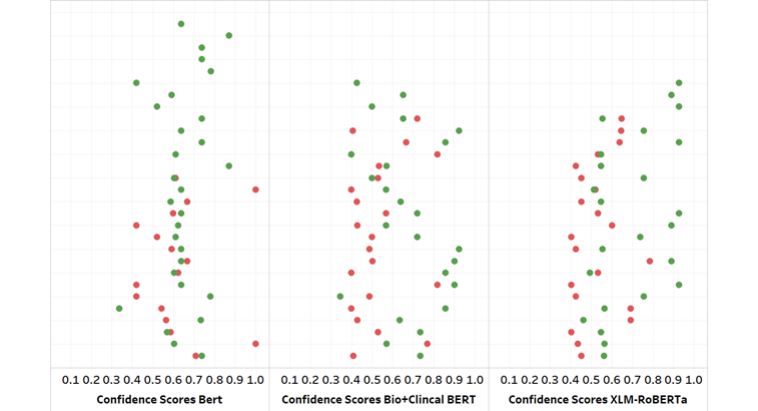
Jitter plot of confidence scores (green=correct predictions and red=incorrect predictions). BERT: Bidirectional Encoder Representations From Transformers; Bio+Clinical BERT: Bidirectional Encoder Representations From Transformers for Biomedical Text Mining+Clinical Bidirectional Encoder Representations From Transformers; XLM-RoBERTa: Cross-lingual Language Model-Robustly Optimized Bidirectional Encoder Representations From Transformers Pretraining Approach.

Figure 2 visually depicts what we discussed in the tables presented in the *Results* section; however, it shows each confidence score for all the studies’ predicted classes for every model. One can see that the green dots (correct predictions) are moving away from the red dots (incorrect predictions) as one studies the models from left to right, indicating higher confidence levels for correct predictions. In conclusion, BERT (the more accurate model) was not able to distinctly give us high or low confidence scores as Bio+Clinical BERT and XLM-RoBERTa did. This presents a trade-off between choosing a model with better predictions but no useful confidence scores and models with more incorrect predictions but more trustworthy confidence scores. Essentially, if we think of this AI system as a fourth reviewer, we want the confidence scores to be an asset in trusting its predictions; for example, a prediction of class 0 with only 40% confidence allows us to use more of our own discretion. However, a model with 60% confidence for every single prediction is not helpful in adjusting our labeling. We want a model that can give high confidence for correct predictions and low confidence for incorrect predictions so that we do not blindly trust a model’s predictions without any threshold regarding its confidence.

### Conclusions

In summary, to classify a study’s potential community partner role, we compared the predictions of 3 transformer-based models with those of 3 domain experts on 45 studies from our university’s IRB database. Our main experiment was to test whether calibrated confidence scores could be an asset in making the AI system more like a fourth reviewer in classifying these studies rather than solely relying on it to make decisions. On the basis of the explication in the *Related Work* subsection, it seems that a measure of confidence can influence humans heavily, but our experiments showed that the more confident a model was on average, the less accurate it became. We believe that this work can stimulate the design and conception of AI systems being used as support for complex decision-making, and although the accuracies of the models were not terrible to begin with, perhaps the use of calibrated confidence scores as a way to influence domain expert decisions sounds too good to be true. Additional improvements can be made, such as figuring out a way to lessen the sampling bias we may have caused when choosing our sample of 45 studies with which to make comparisons, discovering new ways of training our models as well as adding to our training data, making use of the combination of all 3 models’ predictions and confidence as opposed to only choosing those of 1 model, and researching additional techniques that may be better suited for explaining why models make their predictions (ie, local interpretable model-agnostic explanations). In conclusion, identifying CEnR and classifying levels of engagement allow us to help organizations, better serve our stakeholders, and plan for the infrastructure needed to support community engagement. However, our study raises questions about the usefulness of calibrated confidence scores in classifying these studies. The search continues to find a trustworthy algorithmic way of classifying these research studies, which can improve the efficiency and effectiveness of identifying key CEnR metrics.

## Abbreviations

AI: artificial intelligence
AutoML: automated machine learning
BERT: Bidirectional Encoder Representations From Transformers
CEnR: community-engaged research
DL: deep learning
IOED: illusion of explanatory depth
IRB: institutional review board
ML: machine learning
XLM-RoBERTa: Cross-lingual Language Model-Robustly Optimized Bidirectional Encoder Representations From Transformers Pretraining Approach

## Data Availability

The data sets used during analysis are not publicly available because we have no authorization to make them available. The code is publicly available in the form of a GitHub repository [42].
